# Management of pineal and colloid cysts

**DOI:** 10.1136/practneurol-2020-002838

**Published:** 2021-05-26

**Authors:** Michael D Jenkinson, Samantha Mills, Conor L Mallucci, Thomas Santarius

**Affiliations:** 1 Clinical and Molecular Cancer, University of Liverpool, Liverpool, Merseyside, UK; 2 Neurosurgery, The Walton Centre NHS Foundation Trust, Liverpool, Liverpool, UK; 3 Neuroradiology, The Walton Centre NHS Foundation Trust, Liverpool, Liverpool, UK; 4 Paediatric Neurosurgery, Alder Hey Children's NHS Foundation Trust, Liverpool, Merseyside, UK; 5 Clinical and Academic Neuroscience, Addenbrooke's Hospital, Cambridge, Cambridgeshire, UK

**Keywords:** clinical neurology, CSF, headache, neurosurgery, neuroradiology

## Abstract

The widespread use of MRI has led to the increasingly frequent diagnosis of pineal and colloid cysts. While most are small and incidental, do not require long-term monitoring and will never need treatment, they are a cause of patient anxiety and clinician uncertainty regarding the optimal management—particularly for larger cysts or those with an atypical appearance. Occasionally pineal cysts, and more commonly colloid cysts, cause hydrocephalus that requires urgent neurosurgical treatment. More recently the non-hydrocephalic symptomatic pineal cyst has been described in the neurosurgical literature but there is controversy over this entity and its management. This review addresses the difficulties in managing pineal and colloid cysts and provides a pragmatic framework for the practising clinician.

## Introduction

The most common midline intraventricular lesions are pineal and colloid cysts. While they are both histologically benign, can cause hydrocephalus requiring neurosurgical intervention and some aspects of their clinical presentation may overlap, they typically present with very different clinical features and require different management approaches. This review article deals with them as separate entities.

## Pineal cysts

### Epidemiology

Pineal cysts are histologically benign lesions arising from the pineal gland. Hence, they are primarily located in the quadrigeminal cistern with its anterior wall protruding to a variable extent into the third ventricle. Autopsy studies show that microscopic pineal cysts are found in 25%–40% of pineal glands.^1^ Since the advent of MR brain imaging, the incidence of pineal cysts has been reported to vary from 0.58% to 10.8% in large consecutive brain MR imaging studies.^2–4^ Studies in selected populations have reported higher incidence: up to 23% in healthy volunteers^5^ and 11% in children being investigated for short stature or precocious puberty.^6^ The female:male ratio of pineal cysts is approximately 1.7:1.^2–4^ Pineal cysts are most common in the third and fourth decades of life and are infrequent in children aged less than 10 years and in adults aged over 70 years.^2–6^

### Pathophysiology

Several pathophysiological processes are hypothesised to cause cystic enlargement of the pineal gland^2^:

1. Enlargement of an embryonic remnant of the pineal diverticulum within the pineal gland.
2. Formation of a separate glial-lined cyst.
3. Degeneration or necrosis of pineal parenchymal cells.

Histopathological examination of pineal cysts removed at surgery shows that they are typically composed of an inner lining of glial tissue surrounded by an outer rim of pineal gland tissue. Cysts can be simple (unilocular) or complex (multilocular with septations) without an epithelial lining.^7^ While the mechanism that initiates their development is unknown, incidence rates derived from MR studies suggest that the natural history is that pineal cysts form in adolescence, enlarge through young adulthood and then regress in later life. Many pineal cysts become only symptomatic in the third or fourth decade, but it is not clear whether the cysts have developed shortly before presentation or whether the symptoms result from enlargement of a cyst from decades ago.^8 9^

### Clinical presection

The classical textbook description of a symptomatic pineal lesion is obstructive hydrocephalus and Parinaud’s syndrome. While this is the more typical presentation for primary pineal tumours, germ cell tumours and meningiomas, it is unusual for pineal cysts and accounts for <4% of cases in highly selected case series.^10^ Indeed, the vast majority of pineal cysts are asymptomatic and are incidental findings on MRI performed for another clinical indication.

However, there is a growing neurosurgical literature describing so-called ‘symptomatic pineal cysts’ that do not cause hydrocephalus or Parinaud’s syndrome.^7–9 11–16^ Several case series have been published^7–9 11 12 14 16^ that variously ascribe the following symptoms, typically in combination, as being caused by pineal cysts:

1. Paroxysmal headache that is not of a typical migraine pattern (ie, sudden onset, exacerbated by postural changes).
2. Intermittent nausea and/or vomiting.
3. Visual disturbance such as blurred vision, greying of colours, altered visual perception.
4. Transient impaired conscious level.
5. Gait instability.
6. Hypersomnolence.

Whether pineal cysts measuring 10–25 mm in diameter can cause these symptoms is controversial and the cause of most of the symptoms in patients with symptomatic pineal cysts is unknown, although there are several hypotheses. The presumed causative link between pineal cyst symptoms is the fact that most of the symptoms either resolve or improve following surgery. It should be noted that much larger, solid pineal region tumours (eg, pineocytoma) can sometimes be minimally symptomatic.

### Imaging features

**Figure 1:**
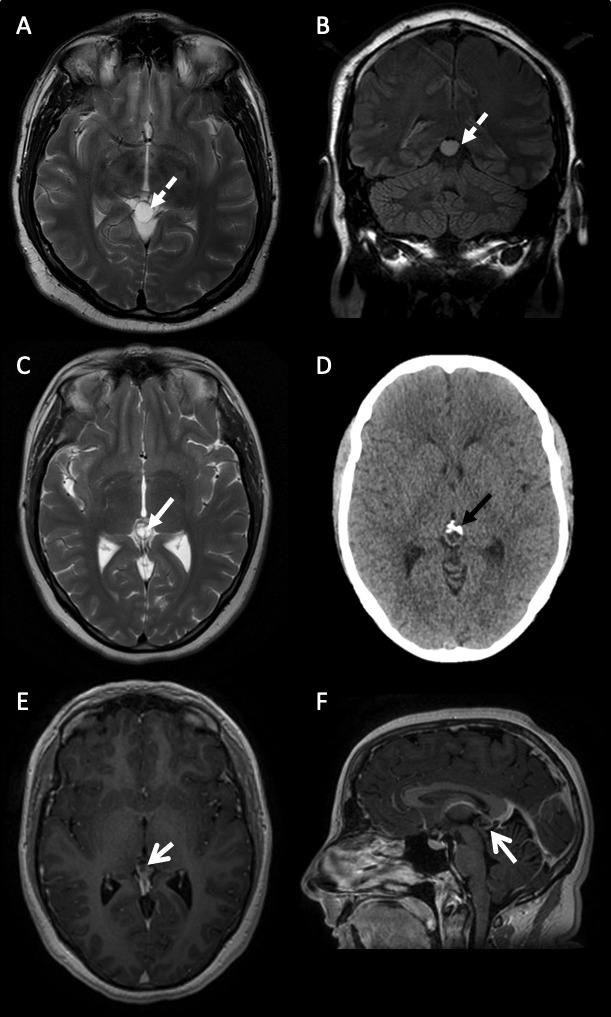
Varying MR brain scan features of pineal region cysts. (A) and (B) A 38-year-old man with a simple pineal region cyst (A) axial T2-weighted and (B) coronal FLAIR sequences of a simple unilocular pineal region cyst with no internal structure (dashed white arrow). The signal hyperintensity does not suppress on FLAIR imaging. (C–F) A 31-year-old woman with an atypical pineal region cyst. (C) Axial T2-weighted sequence of an atypical multilocular, septated (white closed arrow) pineal region cyst. (D) Axial CT scan of head showing anterior calcification (black closed arrow). (E) Axial and (F) sagittal gadolinium-enhanced postcontrast T1 sequences of an atypical septated, posteriorly enhancing (open white arrows) pineal region cyst. FLAIR (Fluid Attenuated Inversion Recovery).

Pineal cysts can be categorised on MR imaging as either simple or atypical.^3^ Simple pineal cysts are unilocular, often with a smooth, thin wall (which may or may not enhance) and can range in size from <5 mm to >25 mm in maximum diameter (figure 1). Atypical cysts are multilocular, have multiple septations and there may also be a solid enhancing component—typically at the posterior part of the cyst in close proximity to the internal cerebral veins (figure 1C–F). Most pineal cysts are isointense with cerebrospinal fluid (CSF) on T1 and T2 but remain hyperintense on FLAIR (Fluid Attenuated Inversion Recovery) due to proteinaceous contents.^3^ Intraoperatively, it is usually indistinguishable from CSF. Occasionally there is calcification with evidence of previous haemorrhage and haemosiderin layering within the cyst. It is important to note that the presence of a solid enhancing component posteriorly is fairly common and does not preclude the diagnosis of a pineal cysts, and indeed when this is used as one of the criteria for surgery (usually when a pineocytoma is considered in the differential diagnosis), the histopathology still confirms a pineal cyst.^7^ In many centres, it is routine practice to image with gadolinium at 12 months for pineal cysts >10 mm diameter although there is no evidence to support this practice and the presence of contrast enhancement does not alter the diagnostic interpretation.^3 4 17 18^

### Management

There are no clinical trials on the treatment of pineal cysts and therefore management recommendations are based on studies of the natural history and neurosurgical outcomes. The management of pineal cysts should consider three clinical scenarios, taking into account the patient’s comorbidity and age:

1. Pineal cysts causing hydrocephalus.
2. Incidental pineal cysts.
3. Non-hydrocephalic symptomatic pineal cysts.

#### Pineal cysts causing hydrocephalus

**Figure 2:**
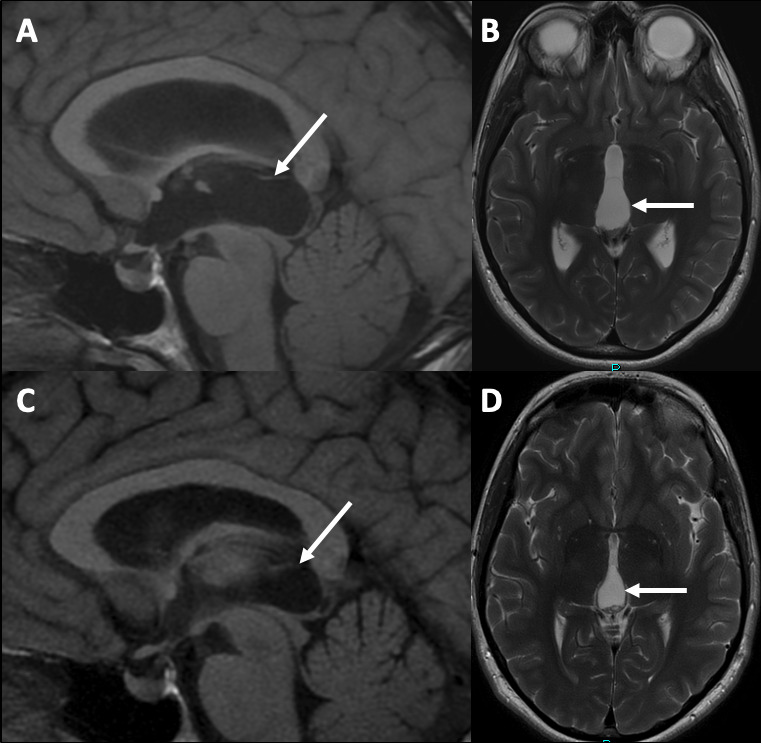
(A) Sagittal T1-weighted and (B) axial T2-weighted MRI sequences showing a large simple pineal region cyst (white arrows) causing symptomatic hydrocephalus at presentation. (C) Sagittal T1-weighted and (D) axial T2-weighted MRI sequences following endoscopic third ventriculostomy and cyst fenestration with collapse of the pineal region cyst and resolution of the hydrocephalus.

These patients typically present to emergency neurosurgery services with raised intracranial pressure. Hydrocephalus may be precipitated by haemorrhage into the cyst and referred to as pineal apoplexy. CSF diversion is required, and this is optimally achieved by an endoscopic third ventriculostomy to relieve the hydrocephalus and endoscopic fenestration of the pineal cyst wall (figure 2). The cyst wall can be biopsied during this procedure, thus providing tissue for diagnosis and if there is continued diagnostic doubt blood; CSF germ cell tumour markers should also be sent. This is valuable especially when patients present with haemorrhage and the diagnosis of a pineal cyst is not straightforward. Follow-up MR imaging with CSF flow studies at 3, 12 and 36 months is required to ensure the endoscopic third ventriculostomy remains patent and functioning. Since the long-term functioning of endoscopic third ventriculostomy in adults is 76%,^19^ if the pineal cysts fenestration remains open then patients can be discharged from routine follow-up, with the caveat to seek medical attention in the future if they develop symptoms of recurrent hydrocephalus. An alternative CSF diversion procedure is insertion of a ventriculoperitoneal shunt, but these have higher failure rates (30%–50%)^20^ than endoscopic third ventriculostomy and are not the preferred treatment of choice. Finally, craniotomy and open resection of the cyst can be undertaken but this is much more invasive and not necessary when lower risk surgical alternatives exist, especially if presenting as an emergency.

#### Incidental pineal cysts

We must not underestimate the psychological impact on individual patients of the discovery of a pineal cyst. Indeed, for any incidental MR finding the clinician must answer two fundamental questions:

1. Is this truly incidental or can any of the symptoms be reasonably attributed to the pineal cysts?
2. Will the cyst change and become symptomatic thus requiring treatment within the patient’s lifetime?

The prevailing opinion and literature support the notion that most pineal cysts are incidental and do not cause symptoms. Pineal cysts may be found on imaging for head injury and tinnitus, as well as for neurological symptoms including new-onset seizures and cerebrovascular disease.^3 4^ Headache accounts for ~50% of the clinical indications for MR scan of brain but in reported series the headache was not attributed to the pineal cyst.^3^

Several large, retrospective cases series have reported the MR appearance of pineal cysts that have been monitored over several years.^3 4 17 18^ A study of MR brain scans in 48 000 adults found 478 incidental pineal cysts and 99% did not grow, highlighting the rationale to discharge with no long-term imaging follow-up.^18^ While several studies^3 4 17 18^ show that cyst size can change on serial MR scans, with up to 6% enlarging and 4% shrinking,^4^ the median change in diameter is only 2.5 mm and does not cause symptoms, and hence routine monitoring is not recommended. Therefore, most patients with truly incidental pineal cysts require only reassurance, and this is best achieved at the first clinical consultation. For most patients, quantifying the risks of surgical intervention (severe neurological deficit and death) versus the natural history is sufficient to reach a shared management decision. Some patients find it reassuring to have a single follow-up interval MR brain scan after 12 months, but this is not mandatory.^14^ Simple pineal cysts below ≤10 mm in diameter do not require any follow-up.

**Figure 3:**
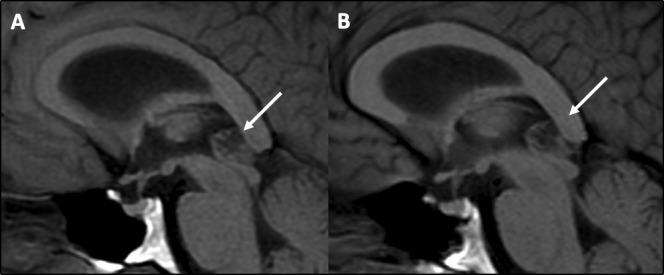
MR scan of brain showing no change in size, shape or appearance on sagittal T1-weighted MRI sequences of an atypical pineal cyst (arrows) in 2016 (A) and 2020 (B).

Due to the potential diagnostic uncertainty in the appearance of atypical pineal cysts (multiloculated, enhancing, solid component) and concern that it may be a pineocytoma, some clinicians may elect to undertake interval MRI every 12 months for 5 years. However, it is worth noting that in the neurosurgical literature, even those pineal cysts with atypical feature (ie, ‘mimicking’ a solid tumour) are histologically confirmed as pineal cysts.^7^ It is the experience of the authors that these atypical pineal cysts also remain stable and therefore do not require longer term follow-up (figure 3).

Adult patients should be reassured that the risk of an incidental pineal cysts enlarging and causing symptomatic hydrocephalus requiring treatment is very low and had not been reported in the literature. In contrast, a recent systematic review of the individual patient data of 109 children over the last 40 years with surgically treated incidental asymptomatic pineal cysts suggests that those with a diameter ≥15 mm are more at risk of progressing to hydrocephalus and visual impairment and interval clinical and MRI follow-up is advised.^13^

#### Non-hydrocephalic symptomatic pineal cysts

This is a growing international literature of undertaking surgery for pineal cysts that are not causing hydrocephalus. In a 2015 survey of 110 neurosurgeons worldwide, 15% responded that they would occasionally operate on patients with so-called ‘non-specific’ symptoms.^21^ In the same year, the first case series of surgical treatment of non-hydrocephalic symptomatic pineal cysts was published^14^ and since then all but one of the subsequent six independent case series^7–9 11 12 16^ showed improvement of symptoms in over 90% patients. In the UK, this is a controversial area and is not part of current standard clinical practice. Surgery in the pineal region is technically challenging and pineal cysts are close to critical neurovascular structures, such as the internal cerebral veins, the tectal plate and its vascular supply. Possible complications from surgery can include venous and arterial haemorrhage or infarction that can affect the tectum, thalamus, temporal and occipital lobes as well as cerebellum with possible clinical consequences including dizziness, hearing loss, various visual problems (including, Parinaud’s syndrome, diplopia and visual field defects), memory, speech and other cognitive impairments, as well as major catastrophe with severe disability and death. Having said that, no serious permanent neurological deficit has been reported among 197 patients from the seven abovementioned cohorts.^7–9 11 12 14 16^

In a series of 28 patients operated between 2007 and 2015, with pineal cysts ranging from 10 to 28 mm diameter, complete resection was achieved in all cases and 27 had complete resolution of their preoperative symptoms. Two patients developed mild headache or discomfort related to scar.^9^ Seventeen of 18 patients operated between 2001 and 2014, with a mean pineal cysts diameter of 15 mm mean diameter reported complete resolution or improved symptoms.^14^ In a series of 60 patients with pineal cysts operated between 1997 and 2015, 19 had surgery for so-called ‘non-specific’ symptoms. In the overall series complete resection was achieved in 58, but eight patients had complications of CSF leak, wound infection or meningitis (13%) and one developed permanent Parinaud’s syndrome (1.6%). All except one had improvement or resolution of their preoperative symptoms.^7^ A systematic review of headache outcomes after surgery for pineal cysts without hydrocephalus reported that out of the 24 cases analysed, 23 had resolution or improvement.^15^ While this list of studies is not exhaustive, it should be noted that based on the published surgical literature^7–9 11 12 14 16^ the number of operated non-hydrocephalus symptomatic pineal cysts is only 1–3 per year in each neurosurgery unit, which is a tiny proportion of all pineal cysts diagnoses.

### Recommendations

While the best practice management of pineal cysts remains to be determined and would benefit from larger comparative clinical studies, the following recommendations constitute a pragmatic approach:

1. Simple pineal cysts of <10 mm diameter do not require any follow-up and can be considered a normal variation of the pineal gland.
2. Adults with incidental pineal cysts (regardless of size) should be reassured and routine follow-up with contrast-enhanced MRI is not usually required.
3. Children with incidental pineal cysts should be monitored as the cysts may enlarge and become symptomatic, with interval follow-up MRI for 1–2 years.
4. Patients with pineal cysts causing hydrocephalus should be treated with combined endoscopic third ventriculostomy and endoscopic cyst drainage (fenestration) and cyst wall biopsy if possible.
5. Atypical pineal cysts (multiloculated, enhancing, solid component) are not primary pineal tumours (eg, pineocytoma) and long-term follow-up is not required. Patients and clinicians may find it reassuring to undertake follow-up MRI at 1, 3 and 5 years and germ cell tumour markers at diagnosis.
6. Patients with suspected non-hydrocephalic symptomatic pineal cysts are rare and probably best referred for evaluation to centres with experience in management of this condition.

### Controversy

Despite the prevailing neurosurgery opinion and consensus worldwide that most pineal cysts are incidental and asymptomatic, there is a growing neurosurgical literature reporting the outcomes of patients undergoing surgical resection to relieve ‘non-specific’ symptoms including headache, sleep, gait, visual and sensory disturbance.^7–9 11 12 14 16^ This raises the question ‘why might some pineal cysts cause symptoms, while other larger cysts, or even tumours, do not, and what is the pathophysiological mechanism?’ Several mechanisms have been proposed, which include direct compression of the tectum, compression of the aqueduct (similar to colloid cysts and foramen of Monro) or crowding of the quadrigeminal cistern consequent compression of the deep venous system.^9^ Given the immediate proximity of visual (superior colliculi, upper tectum, tegmentum anteriorly to the aqueduct), auditory and balance (inferior colliculi, lateral lemniscus), and memory and emotional (habenula, thalamus, tegmentum) infrastructure, as well as more widely distributed functional networks drained by the deep venous system (including the thalami, basal ganglia and its projection fibres, mesial temporal lobes), the causality between pineal cysts and symptoms is not entirely inconceivable, although currently unexplained. Studies relating pineal cysts to sleep disturbance in children^22^ and pineal gland volume to serum melatonin concentrations^23^ provide only limited evidence to support this hypothesis, and do not explain the other so-called ‘non-specific’ symptoms. Clearly, better clinical evidence in the form of prospective cohorts and, ultimately, randomised controlled trials of best conservative versus surgical treatment as well as studies elucidating the pathophysiology behind patients’ symptoms are required.

## Colloid cysts

### Epidemiology

Colloid cysts are rare benign lesion of the third ventricle most frequently located in the vicinity of the foramen of Monro, and account for <1% of all intracranial tumours.^24 25^ They are slightly more common in males than females in a ratio of 1.4:1, and the most present between the third and fifth decades, but they can also be seen in infancy and childhood.^26^ In a meta-analysis of 1278 patients undergoing surgical treatment, the mean age at presentation was 40 years.^27^

### Pathophysiology

Histopathology shows that colloid cysts have a thin collagen wall lined with a single layer of epithelium that may be ciliated or non-ciliated^27^ with mucinous contents that are typically brown-green in colour.^28^ Colloid cysts originate from ectopic endodermal tissue that migrates into the velum interpositum during fetal development.^28^

### Clinical presection

Headache due to raised intracranial pressure and hydrocephalus is the most common presentation and occurs in approximately 60%–90% of patients.^29–31^ The headache is typically frontal, with short exacerbations and may be relieved by sleeping. In more severe cases, patients may be woken by ‘early morning’ headache. It can be associated with nausea and vomiting. Acute hydrocephalus requiring emergency neurosurgery occurs in up to 8% of cases in surgical case series,^32^ is caused by partial obstruction of the foramen of Monro, usually by cysts >1.5 cm in diameter. In the era before CT scanning, diagnosing colloid cysts was challenging and up to 20% presented with sudden death.^33^ However, a recent systematic review found only 107 reported cases and in most of these, patients were actually symptomatic for days, rather than hours, before death.^29^ In fatal cases the mean colloid cysts diameter was 2.0 cm and most were >1.0 cm.^29^ Patients may also have more insidious onset of hydrocephalus associated with worsening short-term and working memory due to either raised intracranial pressure and its effect on the fornices or local compression from the cyst.^34^ Increasingly, colloid cysts are diagnosed as incidental findings due to the widespread use of CT and MR scanning^24 25^ and in these cases there have been no reported cases of sudden death.^35^

### Imaging features

**Figure 4:**
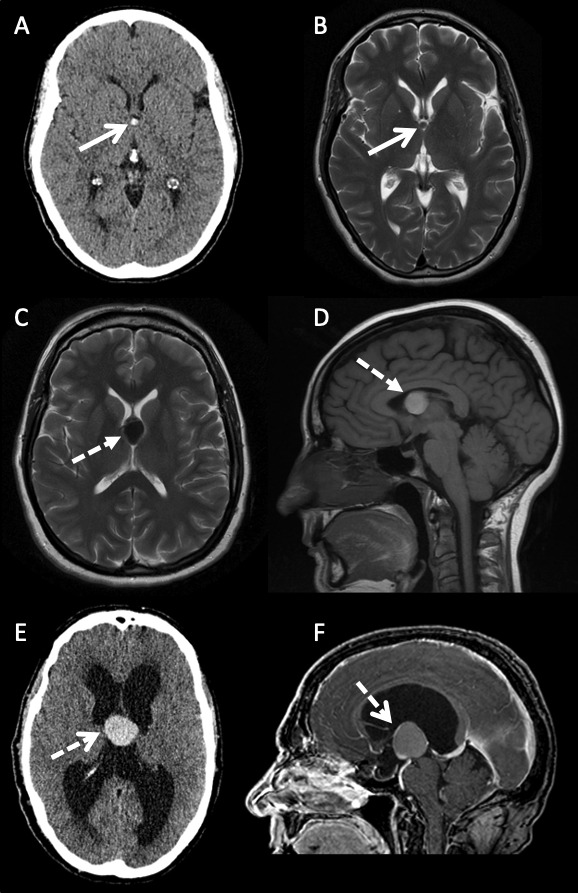
Varying imaging features of colloid cysts. (A) Axial CT scan and (B) axial T2-weighted sequence MRI of a 46-year-old woman with a small CT hyperattenuating (open arrow) colloid cyst within the anterior aspect of the third ventricle. The signal intensity is intermediate to isointense to white matter on the T2-weighted sequence (open arrows). (C) Axial T2-weighted and (D) sagittal T1-weighted MRI sequences of a 50-year-old woman with a large colloid cyst. The lesion is hypointense on T2 and hyperintense on T1 (dashed closed arrow). There is no hydrocephalus. (E) Axial CT and (F) sagittal postgadolinium contrast T1-weighted MRI sequence of a large colloid cyst in a 59-year-old patient presenting with hydrocephalus. The lesion is hyperattenuating on CT, hyperintense on T1 with some peripheral enhancement secondary to displaced and stretched septal veins (dashed open arrows).

Colloid cysts are present in the third ventricle and typically appear as rounded, well-demarcated lesions that range in diameter from a few millimetres to 3–4 cm.^26^ Most are asymptomatic and found incidentally on imaging. Colloid cysts are are best appreciated on CT scanning where they typically appear as hyperattenuating nodules with iso-attenuating or hypo-attenuating cysts rarely seen. There may be ventriculomegaly either with or without periventricular hypoattenuation (figure 4). The appearance on MR imaging is more variable and the T1 and T2 signal characteristics are determined by the cyst contents.^26^ T2 signal can range from markedly hypointense (figure 4C) to isointense to adjacent white matter (figure 4B). T2 hypointense colloid cysts are also low signal on FLAIR imaging making it difficult to identify on this sequence. T1 signal intensity is usually hyperintense to brain parenchyma (figure 4D). Occasionally, there is peripheral rim enhancement due to stretched septal veins (figure 4F).

### Management options

#### Symptomatic colloid cysts

Symptomatic colloid cysts require neurosurgery to prevent neurological deterioration. The goal of surgical treatment is cyst removal and restoration of CSF pathways. Patients who present with acute hydrocephalus causing impaired conscious level or coma require emergency neurosurgery and insertion of bilateral external ventricular drains (or variations such as a single external ventricular drain plus endoscopic pellucidotomy and/or cyst drainage) as a temporising measure before more definitive treatment. There have been many surgical procedures developed, each with their pros and cons.

##### Surgical resection

Surgical resection is the currently favoured approach for colloid cysts, since complete resection is curative and patients do not require long-term follow-up. The surgical challenge is that colloid cysts are deep seated and adjacent to critical neurovascular structures including the fornices and thalamo-striate and internal cerebral veins. There are several surgical techniques including the open microsurgery approach^27 32 36^ and endoscopic resection.^27 30 31^ A large meta-analysis of 1278 patients showed that the complete resection rate was 96.8% with microsurgery and 58.2% with endoscopy, and that re-operation for recurrence was 0.38% for microsurgery and 3.0% for endoscopy.^27^ Although the aim of surgical resection is to restore normal CSF pathways, ventriculoperitoneal shunts were still required in the microsurgery (6.2%) and endoscopy groups (3.9%).^29^ The advantage of open microsurgery is that it can be used for all colloid cysts regardless of location and consistency; however endoscopy is best suited to cases with larger ventricles and cyst contents amenable to aspiration.^31^ A recent systematic review of 962 patients showed higher rates of complete resection with microsurgery (96%) compared with endoscopy (78.5%), but the re-operation rate for symptomatic recurrence was low in both groups (0.33% for microsurgery, 0.61% for endoscopy, p=1.0).^36^ This suggests that endoscopic resection may have a greater role in the management of colloid cysts.^5^ Outcomes from surgery such as relief of hydrocephalus and headache are well reported^37^ but the impact of colloid cysts on neurocognitive function is less well reported. Several small studies have recently shown that cognitive function improves following surgery, but it is not clear whether this is due to removal of the cysts and no direct pressure on the fornices, or just due to resolution of the hydrocephalus.^30 34^

##### Stereotactic drainage of colloid cysts

Frame-based stereotactic aspiration of colloid cysts has fallen out of favour in recent years due to the relatively high rates of failure (10%), incomplete aspiration (23%) and recurrence (40%) and consequent need for long-term follow-up.^38^

##### Insertion of ventriculoperitoneal shunt

A unilateral shunt with fenestration of the septum pellucidum is the least favoured surgical option, even though it is a low-risk procedure. This treatment is used very infrequently but could be reserved for older or frail patients and in cases with unfavourable anatomy with high risk of permanent neurological deficit.

#### Incidental and asymptomatic colloid cysts

**Figure 5:**
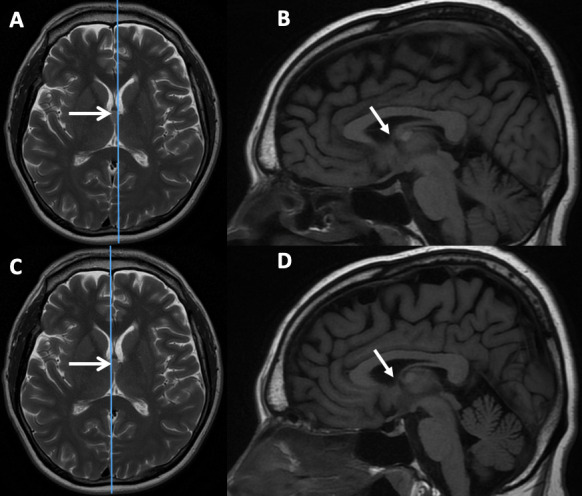
Axial T2-weighted and sagittal T1-weighted MRI sequences showing a colloid cyst (open arrows) arising from the middle part of the third ventricle. The parasagittal cuts to the right (B) and left (D) of midline show that the foramen of Monro is not obstructed (closed arrows) by the colloid cyst, despite the appearance on the T2 axial, therefore the risk of developing symptomatic hydrocephalus requiring treatment is low. The patient is asymptomatic and has been followed for 4 years with no change in MR appearance or clinical features.

As with all incidental imaging findings, the clinician must determine whether any symptoms can be attributed to the identified lesion, which in the case of colloids cysts includes raised intracranial pressure symptoms or cognitive decline. While there is no consensus among neurosurgeons on whether asymptomatic cysts should be treated, the concern about sudden death^29 33^ may result in overtreatment.^32^ The natural history of asymptomatic colloid cysts is less well reported^24 35^ than surgical outcomes. Nevertheless, monitoring has been used for several decades—in a study of 68 patients with a mean cysts size of 8 mm (range 4–18 mm), the incidence of symptomatic progression at 2, 5 and 10 years was 0%, 0% and 8%, respectively—although note that only 14 patients were eligible for evaluation at 10 years.^25^ A systematic review of the natural history of colloid cysts in 176 patients with a median follow-up of 5 years reported that 8.6% underwent surgical intervention and that conservative management was more likely to be offered to older patients with smaller cysts (median diameter 7.5 mm).^35^ This suggests that monitoring asymptomatic cysts is a reasonable approach, and the colloid cyst risk score (table 1) has been proposed^24^ and validated independently.^39^ A colloid cyst risk score of ≥4 is high risk and associated with obstructive hydrocephalus, while a score ≤2 represents a low-risk group.^24^ One of the principal determinants of risk is the anatomical location of the colloid cyst within the third ventricle, since those in the anterior two-thirds are more likely to obstruct the foramen of Monro than those in the posterior one-third (figure 5). There are no studies to guide the frequency of clinical or MRI follow-up for asymptomatic colloid cysts. A pragmatic approach is to offer annual clinical follow-up for 2–3 years and then discharge with red flag advice for the future. This approach provides reassurance to patients, allows any new symptoms to be addressed and reinforces why surgical resection is not the appropriate treatment.

**Table 1:** Colloid cyst risk score has predictive capacity for determining symptomatic clinical status and therefore likelihood of requiring treatment^24^

Criterion	Points
Age <65 years	
Yes	1
No	0
Headache	
Yes	1
No	0
Axial diameter ≥7 mm	
Yes	1
No	0
FLAIR hyperintensity	
Yes	1
No	0
Risk zone	
Yes	1
No	0

FLAIR, Fluid Attenuated Inversion Recovery.

### Recommendations

The best practice management of colloid cysts is determined from retrospective case series and would benefit from larger comparative clinical studies. The following recommendations constitute a pragmatic approach.

1. Patients with symptomatic colloid cysts should be reviewed by a neurosurgeon urgently to consider surgical treatment options.
2. Depending on the clinical urgency, preoperative neurocognitive function should be tested in order to compare to postoperative outcomes.
3. All patients with incidental colloid cysts should be reviewed by a neurosurgeon to ascertain symptoms, consider risk of requiring future treatment, and to discuss conservative and surgical management options.

### Controversies

Although neurosurgery techniques and operative safety have improved massively over the last 20–30 years, the increasing diagnosis of incidental and asymptomatic colloid cysts risks overtreatment of these patients. Increasingly neurosurgeons advocate conservative management and patients can therefore be discharged from routine clinical or MRI monitoring and given advice to seek medical attention if they become symptomatic.

**Key points**

- Large pineal cysts may result in de novo presentation with acute hydrocephalus that requires treatment by cerebrospinal fluid diversion (eg, endoscopic third ventriculostomy and cyst fenestration).
- Symptomatic pineal cysts without hydrocephalus are a rare and relatively new entity; patients with such lesions are best assessed in units with relevant specialist expertise.
- Incidental pineal cysts rarely grow, long-term monitoring is not required and the use of contrast-enhanced MRI does not alter the diagnostic interpretation.
- Colloid cysts typically present with raised intracranial pressure symptoms and cause hydrocephalus that requires surgical treatment (microsurgical or endoscopic resection).
- Incidental colloid cysts can be monitored, although 5%–10% may subsequently require surgical treatment.

**Further reading**

- Gokce E, Beyhan M. Evaluation of pineal cysts with MRI. *World J Radiol* 2018;10:65–77.
- Nevins EJ, Das K, Bhojak M, *et al*. Incidental pineal cysts: is surveillance necessary? *World Neurosurg* 2016;90:96–102.
- O'Neill AH, Gragnaniello C, Lai LT. Natural history of incidental colloid cysts of the third ventricle: a systematic review. *J Clin Neurosci* 2018;53:122–6.
